# Electrical field-induced contractions on *Crotalus durissus terrificus* and *Bothrops jararaca* aortae are caused by endothelium-derived catecholamine

**DOI:** 10.1371/journal.pone.0203573

**Published:** 2018-09-10

**Authors:** Rafael Campos, Alberto Fernando Oliveira Justo, Fabíola Z. Mónica, José Carlos Cogo, Ronilson Agnaldo Moreno, Valéria Barbosa de Souza, Andre Almeida Schenka, Gilberto De Nucci

**Affiliations:** 1 Faculty of Medical Sciences- Department of Pharmacology, University of Campinas (UNICAMP), Campinas, Brazil; 2 Faculty of Biomedical Engineering, Brazil University, Itaquera, Brazil; University of Calgary, CANADA

## Abstract

Endothelium is the main source of catecholamine release in the electrical-field stimulation (EFS)–induced aortic contractions of the non- venomous snake *Panterophis guttatus*. However, adrenergic vasomotor control in venomous snakes such as *Crotalus durissus terrificus* and *Bothrops jararaca* has not yet been investigated. *Crotalus* and *Bothrops* aortic rings were mounted in an organ bath system. EFS-induced aortae contractions were performed in the presence and absence of guanethidine (30 μM), phentolamine (10 μM) or tetrodotoxin (1 μM). Frequency-induced contractions were also performed in aortae with endothelium removed. Immunohistochemical localization of both tyrosine hydroxylase (TH) and S-100 protein in snake aortic rings and brains, as well as in human tissue (paraganglioma tumour) were carried out. EFS (4 to 16 Hz) induced frequency-dependent aortic contractions in both *Crotalus* and *Bothrops*. The EFS-induced contractions were significantly reduced in the presence of either guanethidine or phentolamine in both snakes (p<0.05), whereas tetrodotoxin had no effect in either. Removal of the endothelium abolished the EFS-induced contractions in both snakes aortae (p<0.05). Immunohistochemistry revealed TH localization in endothelium of both snake aortae and human vessels. Nerve fibers were not observed in either snake aortae. In contrast, both TH and S100 protein were observed in snake brains and human tissue. Vascular endothelium is the main source of catecholamine release in EFS-induced contractions in *Crotalus* and *Bothrops* aortae. Human endothelial cells also expressed TH, indicating that endothelium- derived catecholamines possibly occur in mammalian vessels.

## Introduction

Electrical field-induced contractions of isolated aorta of the non-venomous snake *Panterophis gutattus* is insensitive to the voltage-gated sodium channel blocker tetrodotoxin [1], inhibited by adrenergic receptor antagonists and abolished by removal of the endothelium [2]. These findings indicate that the endothelium as the potential source for the catecholamines in response to EFS. Moreover, in *Crotalus durissus terrificus* corpus cavernosum, tyrosine hydroxylase, an enzyme essential for catecholamine synthesis in sympathetic nerve fibers, was only detected in the endothelial cells [3]. In mammalian cells, tyrosine hydroxylase has also been identified in endothelial cells in both bovine aortic endothelial cells and mice superficial femoral arteries [4], indicating that endothelial cells are able to produce catecholamines. In this study, we investigated whether aortae from venomous snakes such as *Crotalus durissus terrificus* and *Bothrops jararaca* would present similar behaviour following EFS. In addition, the presence of tyrosine hydroxylase was assessed by immunohistochemistry in the endothelium of both snake aortae and mammalian (human) vessels.

## Material and methods

### Animals

All experimental procedures using *Crotalus durissus terrificus* and *Bothrops jararaca* were approved by the Institutional Animal Care and Use Committee (CEUA/UNICAMP: 1655–1 and 4722–1) and were performed in accordance with the Ethical Principles for Animal Research adopted by the Brazilian College for Animal Experimentation.

The use of both *Crotalus durissus terrificus* and *Bothrops jararaca* were authorized by the Brazilian Institute for Environment (Sisbio: 18020–1 and 20988–5). *Crotalus durissus terrificus* (body weight: 400–750 g) *and Bothrops jararaca* of either sex (body weight: 300–450 g) were provided by the Serpentarium Center for the Study of Nature at the University of Vale do Paraiba (UNIVAP, São José dos Campos, SP, Brazil) and Butantan Institute (São Paulo, SP, Brazil), respectively.

### Human Paranglioma tissue

The protocol was approved by the Ethics Committee of the State University of Campinas (UNICAMP; Protocol Number 1171/2011). For the sake of precision, the paraganglioma used in this study was resected from the cervical region of a 41-year-old woman in 2013, measured 4.5×3.5 cm and was clinically and histologically classified as benign.

### Chemical and reagents

Acetylcholine, guanethidine, phentolamine, phenylephrine, sodium nitroprusside and tetrodotoxin were purchased from Sigma Aldrich Chemicals Co. (Missouri, USA). Rabbit anti-S100p was obtained from Novocastra/Leica Biosystems (Newcastle, UK). Rabbit anti-TH, chicken anti-TH, goat anti-chicken gamma immunoglobulin (IgG) and a rabbit anti-goat IgG were purchased from Abcam (Cambridge, USA).

### Tissue preparation

The snakes were killed with isoflurane inhalation followed by ketamine (70 mg/kg) administration (intracoelomatic route) and their aortae were removed and immediately placed in Krebs-Henseleit solution at 27°C. Subsequently, aortic rings (3 mm) were obtained and suspended vertically between two metal hooks in 10 mL organ baths containing Krebs- Henseleit solution: (mM) NaCl (118), KCl (4.7), CaCl2 (2.5), MgSO4 (1.2), NaHCO3 (25), KH2PO4 (1.2), glucose (5.6) gassed with a mixture of 95% O2; 5% CO2 (pH 7.4) at 27°C.

### Functional protocols for *Crotalus durissus terrificus* and *Bothrops jararaca* aortic rings

Following the 45 min stabilization period, endothelial integrity was evaluated by assessing acetylcholine (1 μM)-induced relaxation. A relaxation exceeding 80% in a ring pre-contracted with phenylephrine (1 μM) was considered as a signal of endothelial functional integrity. In another set of experiments, the endothelium was removed with the aid of a thin stick. The muscular integrity was assessed by a relaxation induced by sodium nitroprusside (SNP; 1 μM).

*Crotalus durissus terrificus* and *Bothrops jararaca* aortic rings were submitted to electrical-field stimulation (EFS) at 60 V for 30 seconds, subsequently, at 4–16 Hz in square-wave pulses; 0.5 ms pulse width; 0.2 ms delay, using a Grass S88 stimulator (Astro-Medical, RI, USA). EFS-induced contractions were performed in the presence and in the absence of the anti-adrenergic agents phentolamine (10 μM), guanethidine (30 μM) and the sodium-channel blocker tetrodotoxin (TTX; 1 μM) in aortic rings with endothelium-preserved rings (n = 3, for each group). In a separate set of experiments the endothelium was removed with the aid of a thin stick, and the effects of EFS were evaluated.

### Histological and immunohistochemical analysis

Following euthanasia, the *Crotalus* (n = 4) and *Bothrops* (n = 6) aorta samples were collected, fixed in 10% neutral buffered formalin for 24 h at 24o C, dehydrated, embedded in paraffin wax and sectioned at 4 μm. Subsequently, these sections were stained with hematoxylin-eosin (HE) for light-microscopy examination. Additionally, representative tissue sections were immuno-stained for S100 protein (S100p, a neural tissue marker) to investigate the presence of nerve fibers within aortic walls or for tyrosine hydroxylase (TH), using the following primary antibodies: (1) rabbit anti-S100p (polyclonal, Cat#NCL-L-S100p, which reacts with cow, human, chicken, pig, kangaroo, dog, cat, monkey, mouse and rat S-100 protein; Novocastra/Leica Biosystems, Newcastle, UK) at 1:200; (2) rabbit anti-TH (polyclonal, Cat#ab6211, which reacts with mouse, rat, guinea pig and human tyrosine hydroxylase and is predicted to react with chicken, chimpanzee and macaque monkey TH; Abcam, Cambridge, USA) at 1:200, 1:500 and 1:1000; and (3) chicken anti-TH (polyclonal, Cat#ab766442, which reacts with mouse, rat and human TH; Abcam, Cambridge, USA) at 1:500. The rabbit anti-TH antibody was used only in preliminary assays designed to establish the best primary antibody for the detection of this enzyme.

Immunohistochemistry was performed manually. Briefly, the sections were de-paraffinized in xylene and rehydrated in a series of ethanol baths of increasing concentration. They were then incubated in citrate buffer at pH 6.0 (S100p detection) or Tris-EDTA buffer pH 9.0 (tyrosine hydroxylase detection) in a steamer set for 40 min (at approximately 95oC). The sections were then incubated for 1h at 24o C with the above-mentioned primary antibodies. Tissue sections receiving the chicken anti-tyrosine hydroxylase antibody were sequentially incubated with a goat anti-chicken gamma immunoglobulin (IgG), and a rabbit anti-goat IgG, for 1h each, before applying the anti-rabbit IgG detection system (both antibodies were from Abcam (Cambridge, USA). Regardless of the primary antibody, the detection system used was the NovoLink Max Polymer Detection System (Novocastra/Leica Biosystems), following the manufacturer’s instructions, and using diaminobenzidine (liquid DAB, DakoCytomation, Carpenteria, USA) as a chromogen (which renders a brown precipitate at the antibody binding site). Finally, the sections were counter-stained with Ehrlich´s hematoxylin and cover-slipped in Entellan.

Negative controls consisted of the omission of the primary antibody and incubation with the primary antibody diluents (as well as with the secondary antibodies, where applicable).This was performed for all the immunohistochemistry assays to identify any background staining. Furthermore, formalin-fixed, paraffin-embedded *Crotalus* (n = 2) and *Bothrops* (n = 4) brain and a human paraganglioma (n = 1) were used as positive controls for the presence of both antigens (i.e., S100p and TH).

All slides were examined using a trinocular Eclipse 50i microscope (Nikon, Tokyo, Japan) coupled to a 5MP CMOS digital camera (Motic, Hong Kong, China).

### Data analysis

Data are expressed as mean ± standard error of mean (SEM) of the number of experiments. To analyze the pharmacological characterization of EFS-induced contractions, two paired contractions in the presence and absence of antagonists (TTX inclusive) were performed, with the first stimulus being the “control” response. The contractions were quantified in milli-Newtons. Student’s t-test (paired or unpaired depending on the protocol) were used. A p value <0.05 was considered significant.

## Results

### Functional characterization of endothelium-released catecholamine in *Crotalus* and *Bothrops* aortae

**Fig 1:**
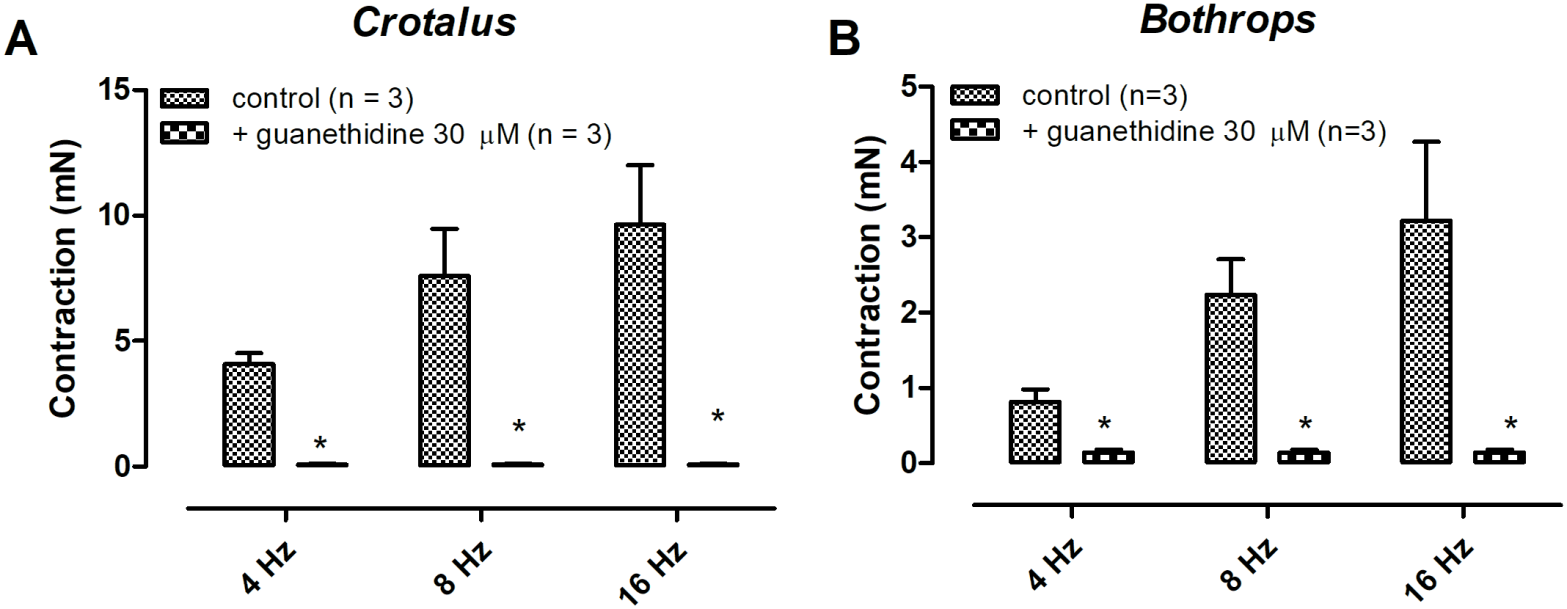
Effect of guanethidine (30 μM) on electrical field stimulation-induced contractions of aortic rings isolated from *Crotalus* (A) and *Bothrops* (B). Data are expressed as mean ± SEM. Paired Student’s t test,*P <0.05 vs control (n = 3, for each group).

**Fig 2:**
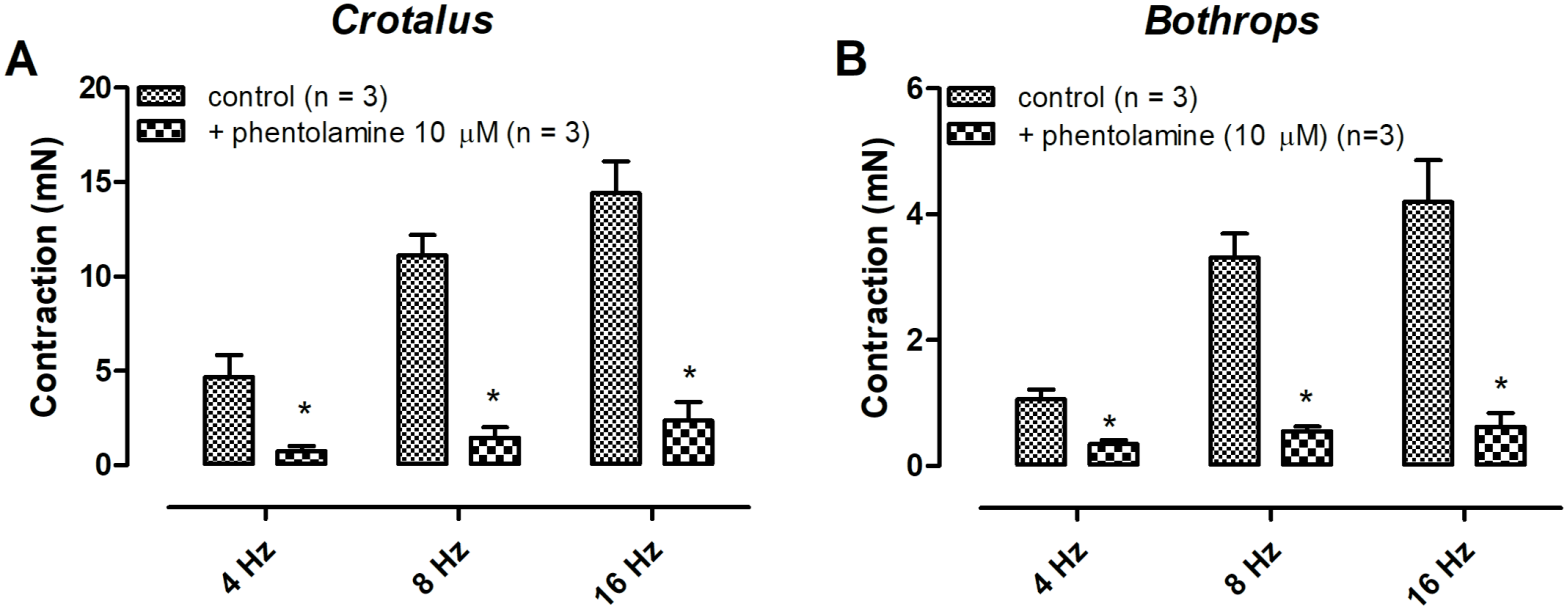
Effect of phentolamine (10 μM) on electrical field stimulation-induced contractions of aortic rings isolated from *Crotalus* (A) and *Bothrops* (B). Data are expressed as mean ± SEM. Paired Student’s t test, *P <0.05 vs control (n = 3, for each group).

**Fig 3:**
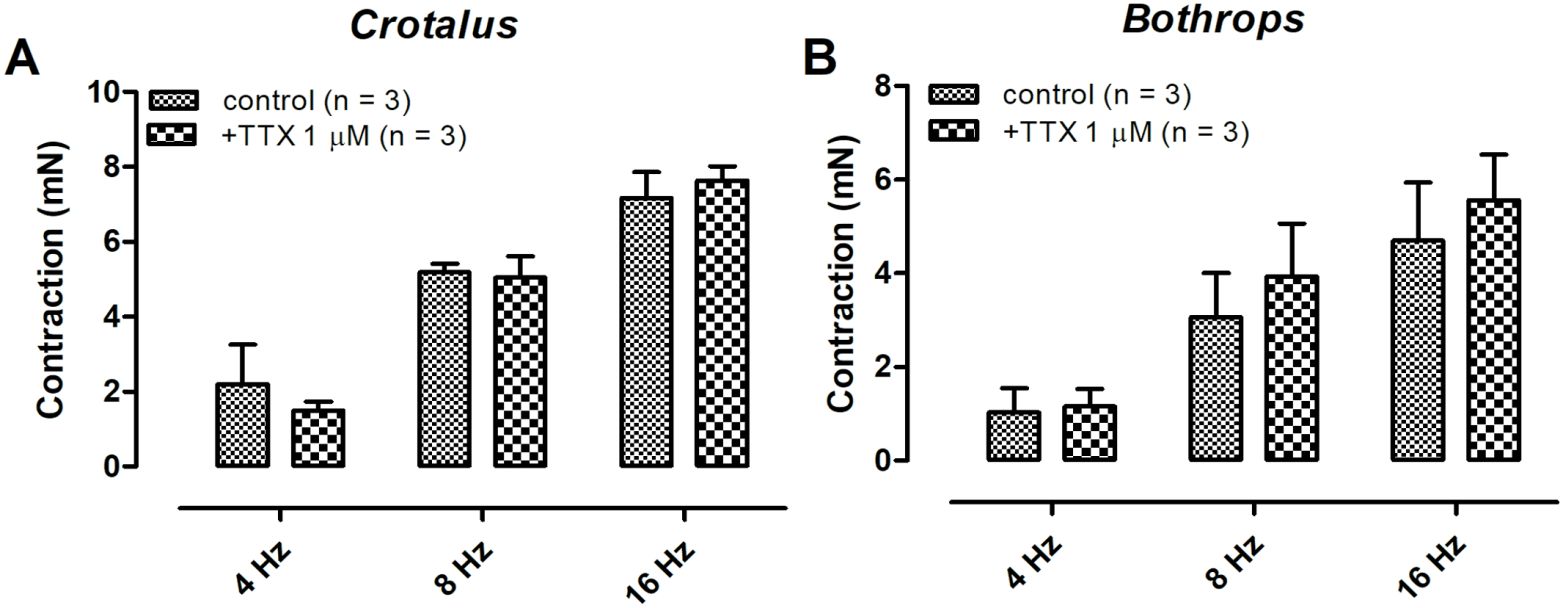
Effect of tetrodotoxin (1 μM) on electrical field stimulation-induced contractions of aortic rings isolated from *Crotalus* (A) and *Bothrops* (B). Data are expressed as mean ± SEM. Paired Student’s t test, *P <0.05 vs control (n = 3, for each group).

**Fig 4:**
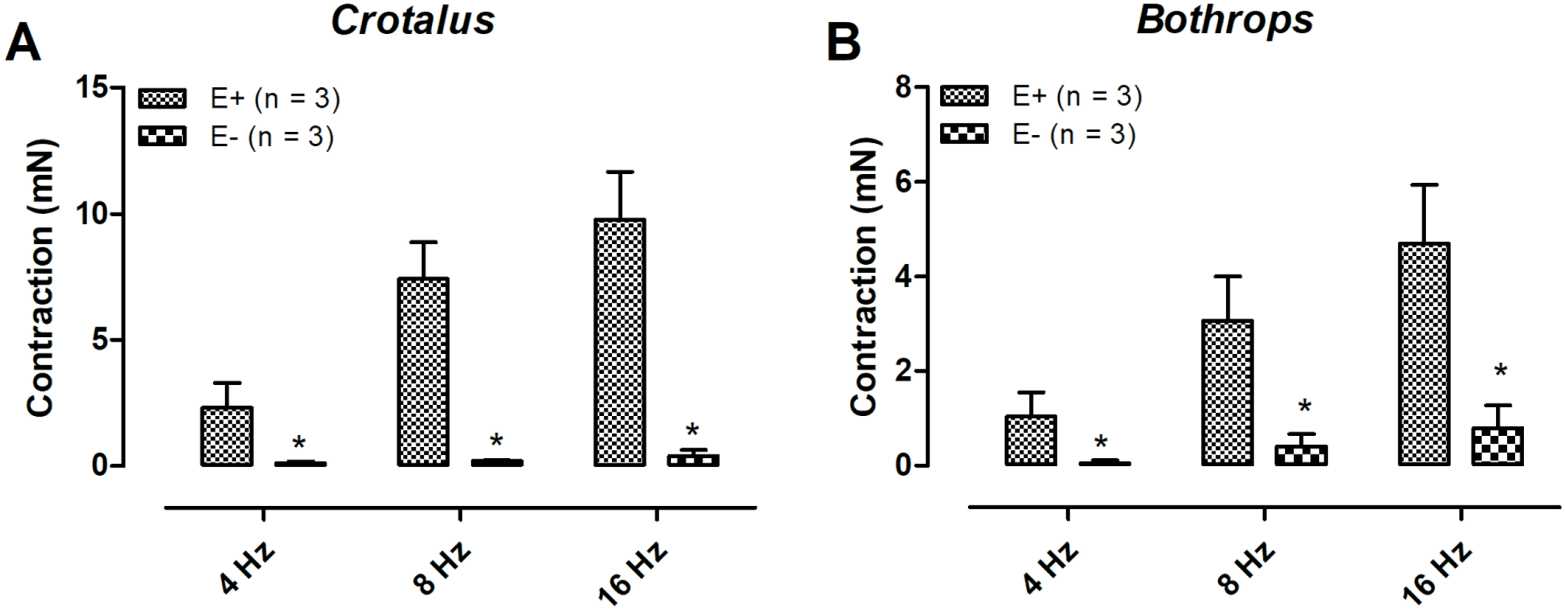
Removal of the endothelium reduced electrical field stimulation-induced contractions in *Crotalus* (A) and *Bothrops* (B) aortic rings. Data are expressed as mean ± SEM. *P < 0.05 vs control (unpaired Student’s t test) (n = 3, for each group).

Electrical field stimulation-induced contractions on both *Crotalus durissus terrificus* and *Bothrops jararaca* aortae were frequency-dependent. Pre- incubation with guanethidine (30 μM, 30 min) abolished the EFS-induced aortic contractions in both animals (Fig 1A and 1B) (n = 3) (p < 0.05). Likewise, pre- treatment with the α-adrenergic antagonist phentolamine (10 μM) significantly reduced the EFS-response in both tissues (Fig 2A and 2B) (n = 3) (p < 0.05). Tetrodotoxin (1 μM) incubation had no effect on the EFS-induced contraction in both aortae (Fig 3A and 3B) (n = 3). Removal of the endothelium abolished the EFS-induced contraction of both aortae (Fig 4A and 4B) (n = 3) (p < 0.05).

#### Immunohistochemistry detection of tyrosine hydroxylase in *Crotalus* and *Bothrops* aortae

**Fig 5:**
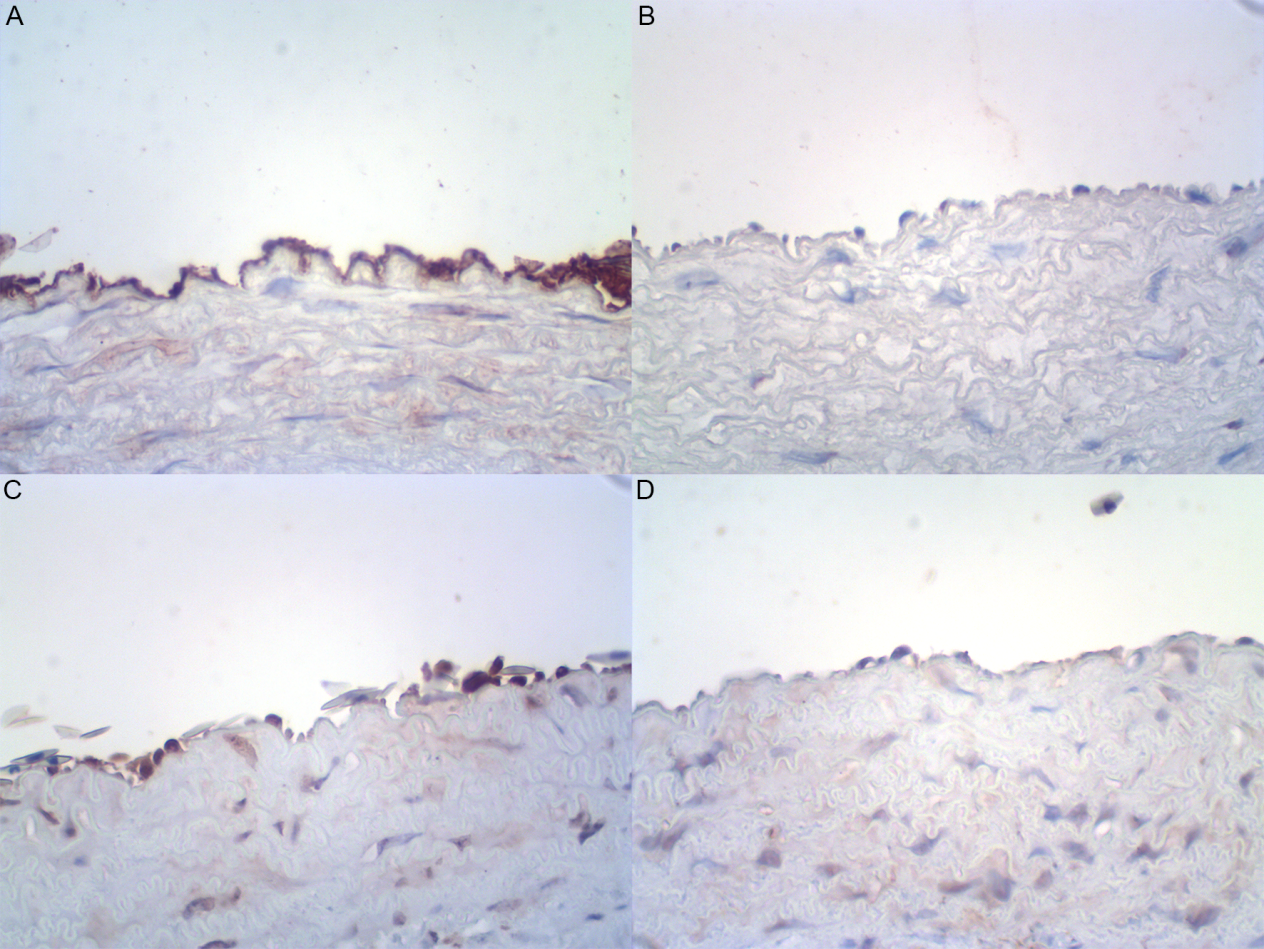
Representative photomicrographs illustrating the presence of tyrosine hydroxylase in endothelial cells of *Crotalus durissus terrificus* (n = 4) **(A)** and *Bothrops jararaca* (n = 6) **(B)** aorta specimens. Lack of S100 protein indicating the absence of nerve fibers within *Crotalus* (n = 4) **(C)** and *Bothrops* (n = 6) **(D)** aortic walls. Immunoperoxidase, 400X (original magnification).

**Fig 6:**
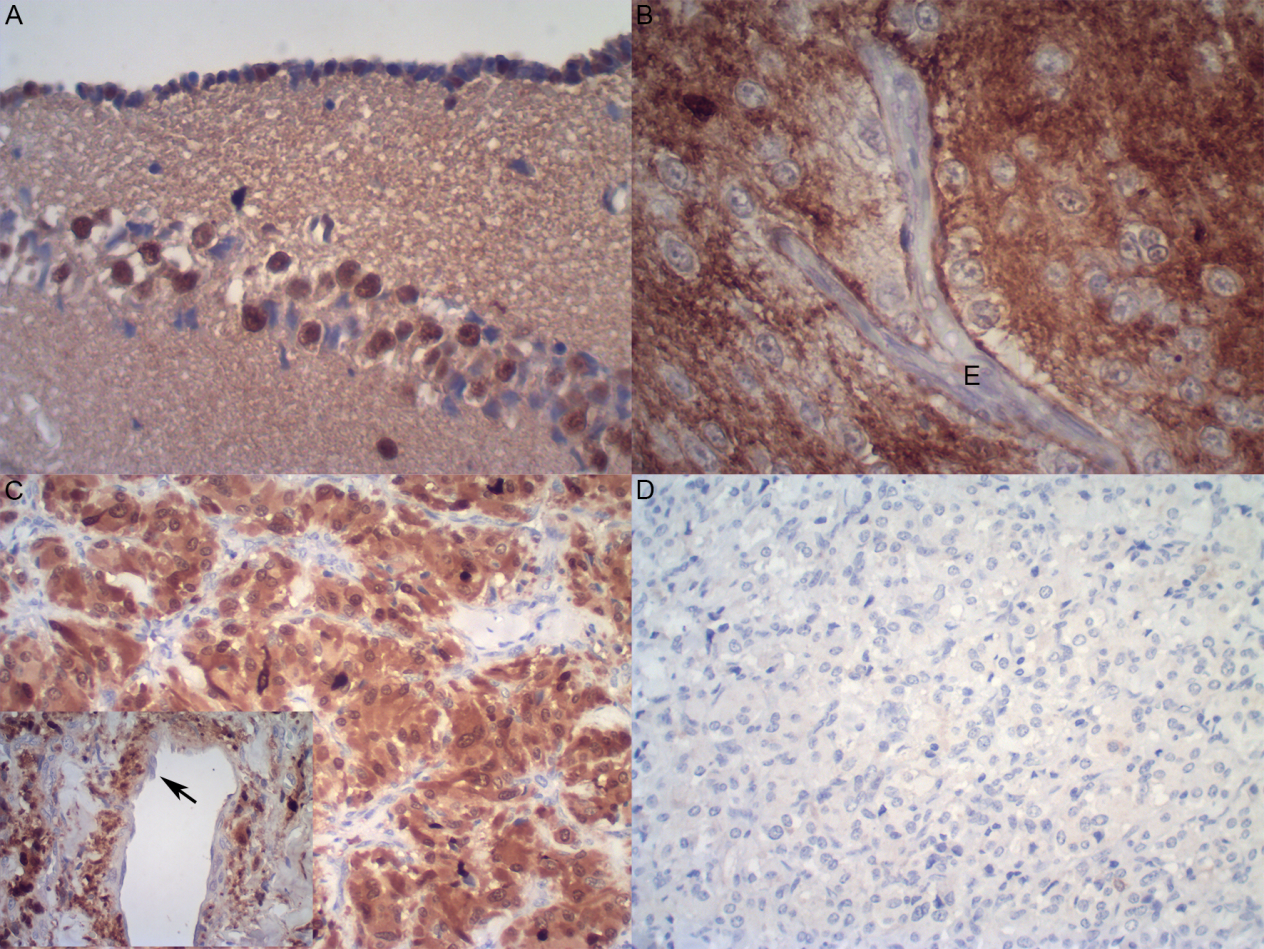
Immunodetection of S100 protein in *Crotalus* (n = 2) **(A)** and *Bothrops* (n = 4) **(B)** central nervous system, as well as in human paraganglioma tissue (n = 1) (positive controls). Notice the lack of S100 protein positivity in endothelial cells from *Bothrops* brain **(B)** and paraganglioma vessels (inset arrow in **C**). No staining was observed in the negative control (omission of primary antibody). Immunperoxidase, 400x (**A** and **B**), 200x (**C**, including inset, and **D**), original magnifications.

**Fig 7:**
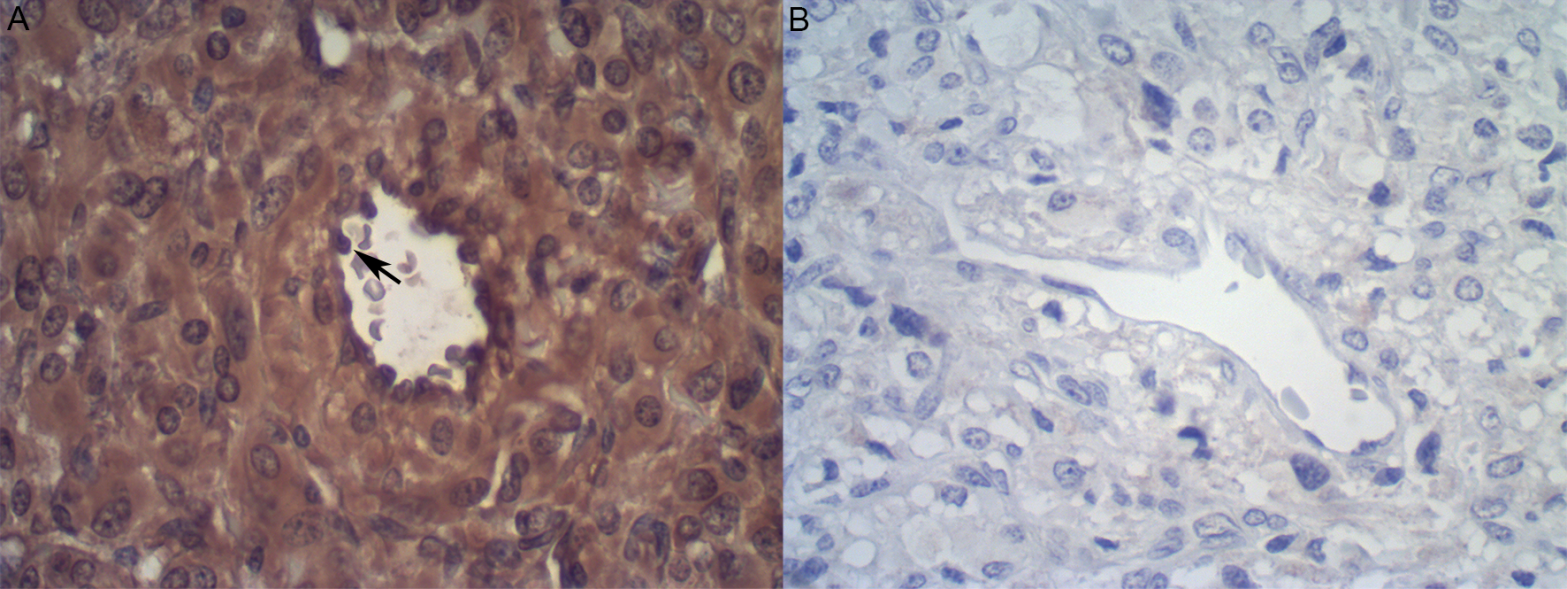
**A**: Immunodetection of tyrosine hydroxylase in human paraganglioma tissue (n = 1) (positive control). Notice the presence of S100 protein in both tumour and endothelial cells (arrow) (n = 1). **B**: no staining is observed in the negative control (omission of primary antibody). Immunoperoxidase, 400x original magnification.

The results concerning S100p and tyrosine hydroxylase immunodetection in aortic specimens of *Crotalus durissus terrificus* are summarized in Table 1 and Figs 5–7. S100p was consistently negative in all aortic tunicae from both snakes investigated (4 out of 4 stained specimens), indicating the absence of nerve fibres in this vascular structure (Table 1 and Fig 5). As expected, in both positive controls (i.e., in human paraganglioma tissue and in snakes’ brains), S100p was diffusely positive (Fig 6). The presence was strongest in the nuclei/cytoplasm of paraganglioma cells (Figs 6 and 7). No immunostaining was observed in stromal cells (such as smooth muscle cells, fibroblasts or endothelial cells) from either positive control tissues (Figs 6 and 7).

**Table 1:** S100 protein and tyrosine hydroxylase immunodetection in *Crotalus* and human tissues: Frequency of positive cases and immunostaining intensity.

Antibody (dilution)	Aorta, *Crotalus* *Frequency*^a^ *(Intensity*^b^*)*	Brain, Crotalus *Frequency*^a^ *(Intensity*^b^*)*	Paraganglioma, Human *Frequency*^a^ *(Intensity*^b^*)*
Rabbit polyclonal anti-S100p (1:200)	0/4 (-)	2/2 (+-++)	1/1 (+++) in neoplastic cells
Rabbit polyclonal anti-tyrosine hydroxylase^c^ (1:200, 1:500, 1:1000)	2/2 (+-+++) in tunica intima/vasa vasorum (endothelium) and tunica media (BS in collagenous elements)	NA	1/1 (+-+++) in neoplastic and endothelial cells
Chicken polyclonal anti-tyrosine hydroxylase (1:500)	4/4 (+-+++) in tunica intima/vasa vasorum (endothelium)	2/2 (+-++) in neuron cell bodies of the cortex and (+) in neuropile	1/1 (+-+++) in neoplastic and endothelial cells

^a^Frequency: no. of positive samples/total no. of samples.

^b^Immunostaining intensity scale: (-): negative; (+): weak staining; (++): moderate staining; (+++): strong staining.

^c^Performed only in development/standardization assays.

BS: background staining. NA: non-applicable (immunostain was not performed).

In preliminary experiments (development/standardization assays), tyrosine hydroxylase was detected in all tested tissues (i.e., in the human paraganglioma and in aorta samples from two *Crotalus* serpents) using both primary antibodies (that is, the rabbit- and the chicken-raised anti-TH antibodies). Tyrosine hydroxylase was consistently found in the cytoplasm of paraganglioma neoplastic cells and of the endothelia from both tested tissues (Figs 5 and 7). However, while both antibodies resulted in similar immunostaining intensities in the mentioned cell types, the one raised in chicken was considered more specific because it was not associated with significant background (nonspecific) staining in collagenous components of the aortic tunica media (as opposed to the rabbit anti-TH antibody) (data not shown). Therefore, the chicken anti-TH antibody was selected and subsequently applied to all tissue samples tested in this study.

Using the chicken anti-TH monoclonal antibody, TH was found to be moderately to strongly positive in paraganglioma neoplastic cells and endothelia (Fig 7), in cortical neurons from the positive control *Crotalus* brains (data not shown) and, most importantly, in aortic endothelial cells (Figs 5 and 6). Notice that the latter cells comprise not only the endothelial cell lining of the aorta lumen, but also the endothelia observed in the vasa vasorum.

In the paraganglioma sections, TH presence was observed in the endothelia of small to medium-sized vessels, which were seen either in close proximity to the tumour cells (small, thin-walled, venule-like vessels) or embedded in the tumour fibrous septae (larger, vein-like vessels, occasionally bearing an irregular/incomplete smooth muscle layer) (Fig 7). Similar tyrosine hydroxylase and S100p detection was also observed in *Bothrops* aortae and brain tissues (Table 2 and Fig 5).

**Table 2:** S100 protein and tyrosine hydroxylase immunodetection in *Bothrops* tissues: Frequency of positive cases and immunostaining intensity.

Antibody (dilution)	Aorta, *Bothrops* *Frequency*^a^ *(Intensity*^b^*)*	Brain, *Bothrops* *Frequency*^a^ *(Intensity*^b^*)*
Rabbit polyclonal anti-S100p (1:200)	-	4/4 (++-+++) specimes Mostly in: glial cells (cytoplasmic and nuclear), ependymal cells (cytoplasmic and nuclear) and neuropil
Chicken polyclonal anti-tyrosine hydroxylase (1:500)	6/6 (+-+++) Mostly in: luminal (+6/6) and vasa vasorum (+2/2) endothelia	4/4 (+-+++) Mostly in: neurons (cytoplasmic) and neuropil

^a^Frequency: no. of positive samples/total no. of samples.

^b^Immunostaining intensity scale: (-): negative; (+): weak staining; (++): moderate staining; (+++): strong staining.

## Discussion

Our results clearly demonstrate that the EFS-induced contractions of both *Crotalus durissus terrificus* and *Bothrops jararaca* isolated aortic rings were dependent upon catecholamine release, insensitive to tetrodotoxin and abolished by the removal of endothelium. These results confirm previous results obtained in isolated aortic rings of *Panterophis guttatus* (a non-venomous snake), reinforcing that the endothelium is the source for catecholamine release. Indeed, in *Crotalus durissus terrificus* corpus cavernosum, the EFS-induced contraction was dependent upon catecholamine release, but the enzyme tyrosine hydroxylase was identified only in the endothelium [3].

Immunohistochemistry for tyrosine hydroxylase of both *Crotalus durissus terrificus* and *Bothrops jararaca* aortae revealed that the enzyme is present in the endothelial cells. The antibody used for the immunohistochemistry was an anti-chicken tyrosine hydroxylase antibody. Tyrosine hydroxylase has not been cloned in reptiles. Using an avian antibody in a different taxon is complex, but the pharmacological findings support the evidence for the presence of this enzyme in snake endothelial cells. Furthermore, the immunohistochemistry was also positive using a mammalian antibody (anti-rabbit tyrosine hydroxylase antibody). As expected, the immunohistochemistry for tyrosine hydroxylase was positive in both *Crotalus durissus terrificus* and *Bothrops jararaca* brains as well as in the human paraganglioma sample.

Tyrosine hydroxylase is the enzyme responsible for the conversion of tyrosine to L-dihydroxyphenylalanin (L-DOPA), the precursor of dopamine [5]. Synthesis of adrenergic catecholamines has been shown to occur in bovine aortic endothelial cells and in mouse femoral arteries [4]. Our results identify, for the first time, immunoreactivity of TH in mammalian (human) endothelial cells in a neuroendocrine neoplasm. This raises an interesting possibility of a potential role of endothelial-derived catecholamines modulating tumour growth. Indeed, propranolol (a non-selective β-antagonist) administration in patients with ovarian cancer reduced the tumour progression [6]. Propranolol administration is also related to reduction of death risk in patient with malignant melanoma [7,8] reduction in breast cancer progression and mortality [9,10,11] and improved the lung survive outcomes in patient with non-squamous lung cancer treated with radiation therapy [12]. However, the physiological and pathophysiological role of endothelial-derived catecholamines in both circulation control and cancer progression remains to be further investigated.

## Conclusion

Our results indicate that endothelial-derived catecholamines may modulate snake vascular tonus and confirms that human endothelial cells express tyrosine hydroxylase.
